# Morbihan disease-like presentation of tuberculoid leprosy

**DOI:** 10.1016/j.jdcr.2023.12.012

**Published:** 2024-01-01

**Authors:** Abdullah Al-Omair, Moath Al Busair, Abdulaziz Al Sadhan, Ahmed Alhumidi

**Affiliations:** a Department of Dermatology, Prince Sultan Military Medical City, Riyadh, Saudi Arabia; b Department of Dermatology, Security Forces Hospital, Riyadh, Saudi Arabia; c Department of Pathology, College of Medicine, King Saud University, Riyadh, Saudi Arabia

**Keywords:** isotretinoin, leprosy, Morbihan disease, rosaceous lymphedema

## Introduction

Morbihan disease (MD), also known as solid facial edema, is a rare condition characterized by lymphedema or erythematous edema. MD typically affects the middle and upper thirds of the face (the malar regions, nose, glabella, eyelids, and forehead).^1^ This disease can lead to distortion of the facial contour and vision impairment because of a narrowed visual field, which can cause psychosocial stress.^2^

Although the pathogenesis of MD is unclear, previous theories have suggested that it is an end-stage complication of rosacea and acne resulting from impaired lymphatic drainage. However, MD occurs in patients without a history or symptoms of rosacea. Moreover, newer but possible pathogenic factors have been suggested, such as the local dysregulation of lymphatic vessels and lymphatic obstruction by granulomas and histiocytes, which destroy the supporting connective tissue around the dermal lymphatic vessel.^3^ Diagnosis is usually based on supporting clinical and histologic features that are not specific to MD. Therefore, it is essential to exclude other conditions such as orofacial granulomatosis, sarcoidosis, lupus vulgaris, pseudolymphoma, foreign-body granuloma, and granuloma faciale.^4^

We report a 40-year-old man who presented with clinical symptoms of MD as an early presentation of tuberculoid leprosy, which responded well to isotretinoin.

## Case report

A 40-year-old man, with no prior history of acne or rosacea presented to our dermatology department with a chief complaint of bilateral periorbital edema prominent during the morning time that had started 4 months prior, progressively worsened over the same period, associated with painless erythema that was mainly over the right side and right-side epiphora. The patient denied any pain, itching, or worsening of symptoms after exposure to the sun. Extensive laboratory tests including complete blood count, comprehensive metabolic panel, thyroid function, antinuclear antibody, and C3/C4 were performed, and the results were negative or within normal ranges. Moreover, computed tomography revealed severe edema but no other abnormalities.

**Fig 1:**
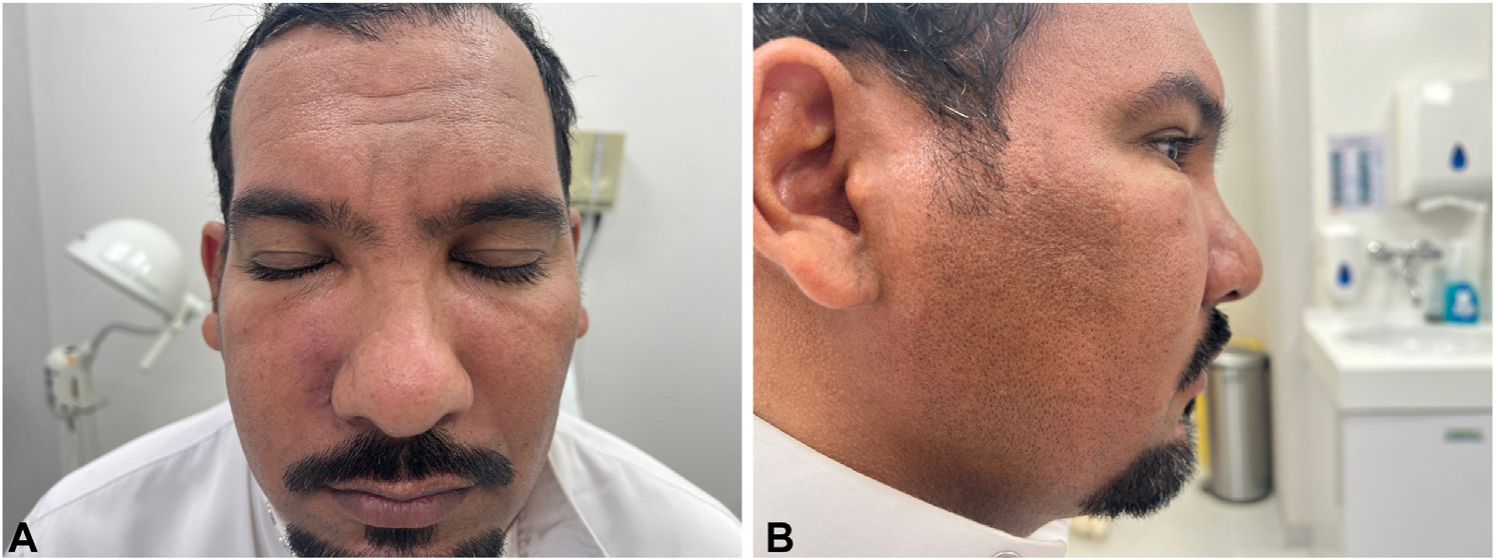
**A,B,** There is ill-defined faint erythema associated with marked periorbital swelling over the right cheek more than the left side. The patient’s eyebrows and eyelashes were preserved, and motor and sensory functions were intact on neurologic examination.

During the initial examination, ill-defined faint erythema associated with marked solid periorbital swelling was observed on the right cheek more than that on the left side and along the nose, glabella, and forehead. At first presentation, the patient’s eyebrows and eyelashes were preserved, and motor and sensory functions were intact on neurologic examination (Fig 1, *A* and *B*).

**Fig 2:**
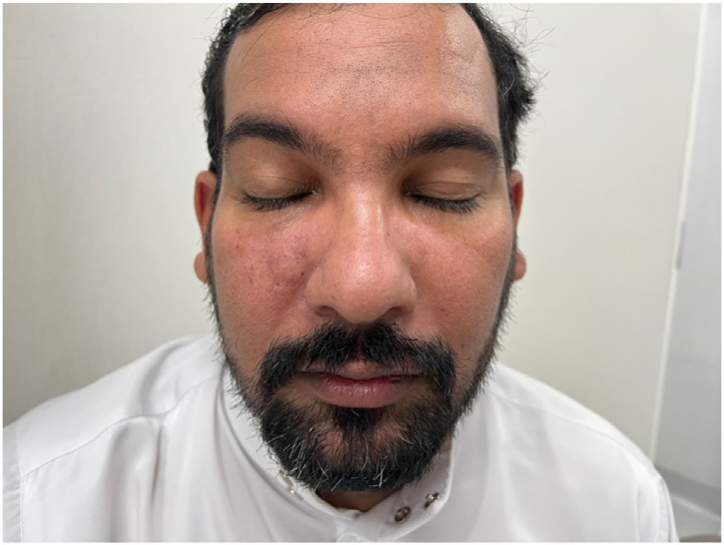
There is solitary 2 × 2 cm annular erythematous plaque raised border with central clearing on right perinasal surface.

A clinical diagnosis of MD was made based on the patient’s symptoms and signs and oral isotretinoin (40 mg) was initiated once daily. After 2 months of treatment, the swelling over the face markedly improved. However, the patient experienced a pricking sensation over the right cheek with a persistent erythematous lesion in the same area. A solitary 2 × 2 cm annular erythematous plaque with a raised border and central clearing on the right perinasal surface was detected. Upon neurologic examination, the patient exhibited diminished sensation over this particular region, while maintaining full-body motor and sensory functions without any observable additional cutaneous features (Fig 2).

**Fig 3:**
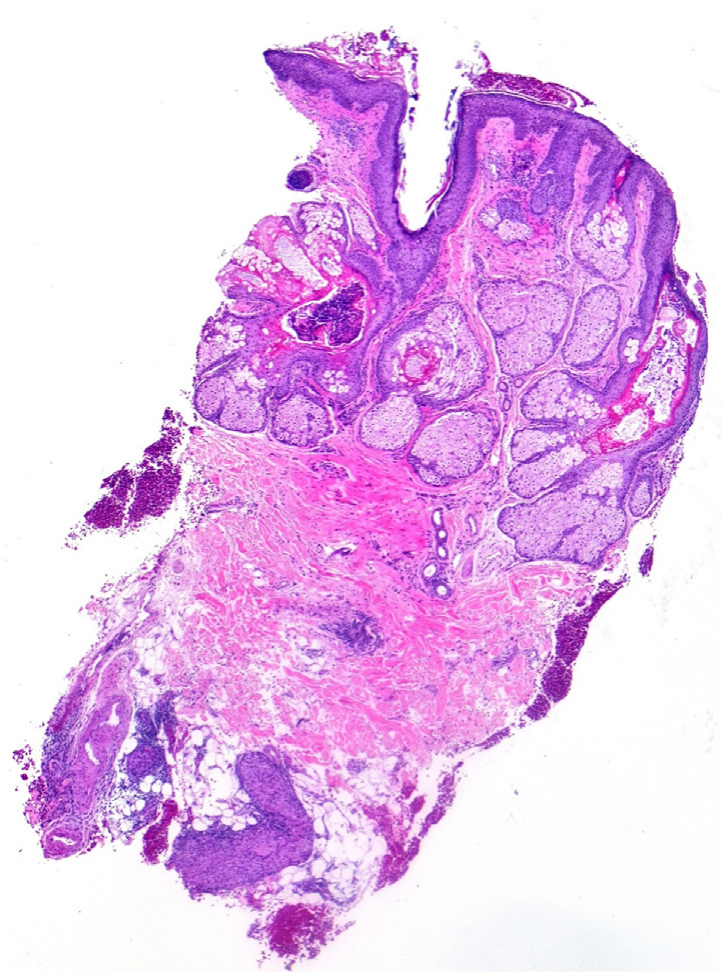
Photomicrograph of skin punch biopsy revels deep granuloma in the subcutaneous fat (hematoxylin-eosin stain; original magnification: ×40).

**Fig 4:**
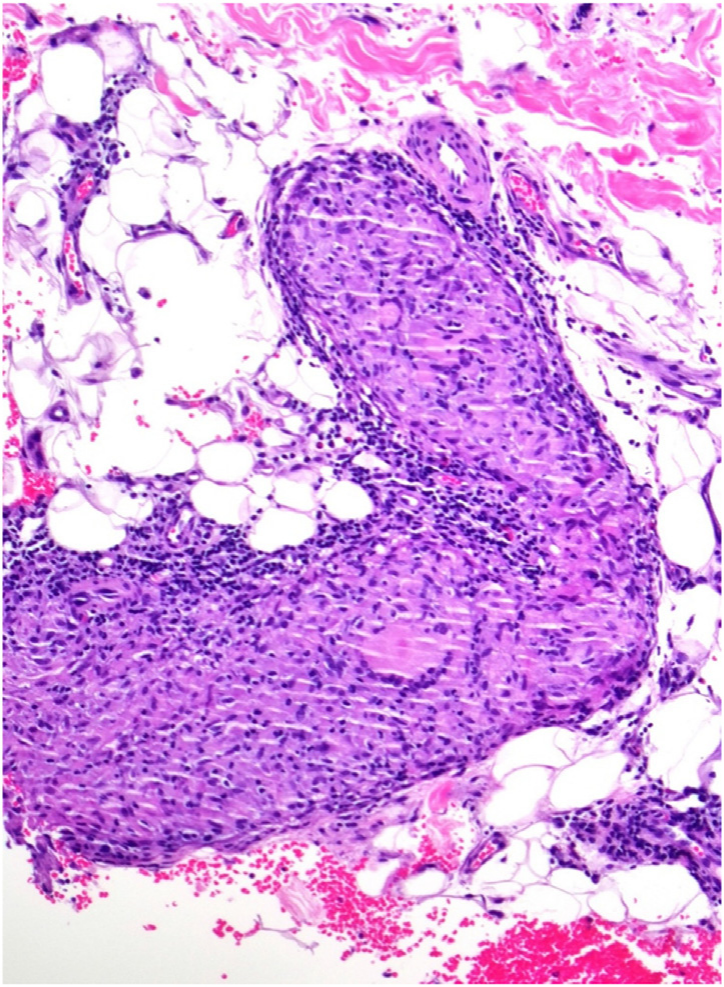
Higher power of the skin biopsy shows collections of epithelioid histocytes around neurovascular bundle (hematoxylin-eosin stain; original magnification: ×400).

Microscopic examination of the area after staining with hematoxylin and eosin showed mild perifollicular lymphocytes with dermal edema, with underlying deep dermal and subcutaneous tissue infiltration of dense collection of epithelioid histocytes that were heavily involved around the neurovascular bundle; however, the Fite-Faraco stain was negative (Figs 3 and 4).

Upon further examination it was revealed that the patient’s mother had a family history of nasal tuberculoid leprosy, which was treated 3 years before the patient’s condition. Therefore, a diagnosis of tuberculoid leprosy was determined based on a based on clinical-pathologic correlation.

## Discussion

Leprosy is a chronic infection caused by *Mycobacterium leprae*. The clinical manifestations of leprosy primarily involve the skin and the nervous system. It is characterized by 2 polar forms: tuberculoid or paucibacillary leprosy (few lesions and a competent immune system) at one end of the spectrum, and lepromatous or multibacillary leprosy at the other end (numerous lesions and a deficient immune system).^5^ Dermal granulomatous infiltrates are observed in the tuberculoid pattern of leprosy. In our case, the linear appearance of the granulomas on histology can be attributed to nerve involvement that is characteristic of tuberculoid leprosy (Fig 4). Additionally, lymphocytes surround epithelioid and Langhans giant cells, the cutaneous nerves can be edematous, and there is an absence of organisms, even with special stains.

The etiopathogenesis of MD is not fully understood. According to a previous study, this is a clinical variation or complication of acne or rosacea.^6^ In our patient, age, clinical picture, and histopathologic results supported a correlation with rosacea, however, the additional presence of linear granuloma along the nerve was consistent with the diagnosis of leprosy. Moreover, the histopathologic characteristics of MD have been poorly described in the literature, with nonspecific findings which typically include edema in the dermis and dilated blood vessels, lymphocytes, neutrophils, perivascular, and perifollicular histiocytes, perifollicular fibrosis, and rarely, an increase in the number of mast cells.^2^^,^^7^ Whether tuberculoid leprosy induces the development of MD or whether the resulting tuberculoid leprosy granuloma leads to lymphatic obstruction with the subsequent development of MD-like features still needs to be adequately elucidated.

As confirmed in the literature, treatment is challenging, and the evidence base is very limited. Difficulties in diagnosis and treatment may be attributed to a poor understanding of disease pathophysiology. Although approximately 20% of patients do not respond to oral isotretinoin, it is considered first-line therapy. However, our patient showed a significant improvement with isotretinoin alone. Other studies have reported variable outcomes with combinations of isotretinoin with ketotifen or clofazimine, prednisolone with metronidazole and ketotifen, and doxycycline with prednisolone.^8^^,^^9^

In conclusion, we present the case of a healthy 40-year-old man with symptoms and signs of MD, which was managed with isotretinoin therapy, triggered by the early presentation of tuberculoid leprosy. Further large-scale studies are needed to determine the relationship between MD and other granulomatous disorders, including tuberculoid leprosy.

## Conflicts of interest

None disclosed.

## References

[1] Heibel H.D., Heibel M.D., Cockerell C.J. (2020). Successful treatment of solid persistent facial edema with isotretinoin and compression therapy. JAAD Case Rep.

[2] Cabral F., Lubbe L.C., Nóbrega M.M., Obadia D.L., Souto R., Gripp A.C. (2017). Morbihan disease: a therapeutic challenge. An Bras Dermatol.

[3] Kim J.H. (2021). Treatment of Morbihan disease. Arch Craniofac Surg.

[4] Kwok C. (2015). Morbihan disease—challenges in diagnosis and management. J Am Acad Dermatol.

[5] Chen K.H., Lin C.Y., Su S.B., Chen K.T. (2022). Leprosy: a review of epidemiology, clinical diagnosis, and management. J Trop Med.

[6] Veraldi S., Persico M.C., Francia C. (2013). Morbihan syndrome. Indian Dermatol Online J.

[7] Mayur O., Martinez R., McNichol M.C., McGee J.S. (2023). Clinical and histological features and treatment outcomes of patients with Morbihan disease: a systematic review. Arch Dermatol Res.

[8] Gil F., Aranha J., Andrade I. (2022). Significant and sustained response with short cycle of low-dose isotretinoin in Morbihan disease. Skinmed.

[9] Guimarães M.J., Lopes A.G., Vieira A.P. (2023). Morbihan disease: a diagnostic and therapeutic challenge. Acta Med Port.

